# Symptom Burden in Long-Term Survivors of Head and Neck Cancer: Patient-Reported Versus Clinical Data

**DOI:** 10.5334/egems.271

**Published:** 2019-07-10

**Authors:** Gaia Pocobelli, Rebecca Ziebell, Monica Fujii, Katherine A. Hutcheson, Steven Chang, Jennifer B. McClure, Jessica Chubak

**Affiliations:** 1 Kaiser Permanente Washington Health Research Institute, US; 2 MD Anderson Cancer Center, US; 3 Josephine Ford Cancer Institute, Henry Ford Health System, US

**Keywords:** head and neck cancer, symptoms, administrative data, accuracy, electronic medical record

## Abstract

**Introduction:**

The symptom burden faced by long-term head and neck cancer survivors is not well understood. In addition, the accuracy of clinical data sources for symptom ascertainment is not clear.

**Objective:**

To 1) describe the prevalence of symptoms in 5-year survivors of head and neck cancer, and 2) to evaluate agreement between symptoms obtained via self-report and symptoms obtained from clinical data sources.

**Methods:**

We recruited 5-year survivors of head and neck cancer enrolled at Kaiser Permanente Washington (n = 54). Symptoms were assessed using the MD Anderson Symptom Inventory head and neck cancer module. For each symptom, we assessed the agreement of the patient’s survey response (“gold standard”) with the 1) medical chart and 2) administrative health care claims data. We computed the sensitivity, specificity, positive predictive value (PPV), and negative predictive value, along with their 95 percent confidence intervals, for each clinical data source.

**Results:**

Eighty percent of patients responded. Nearly all participants (95 percent) reported experiencing at least one symptom from the MDASI-HN, and 93 percent reported two or more symptoms. Among patients reporting a given symptom, there was generally no evidence of the symptom from either clinical data source (i.e., sensitivity was generally no greater than 40 percent). The specificity and PPV of the clinical data sources were generally higher than the sensitivity.

**Conclusion:**

Relying only on medical chart review and/or administrative health data would substantially underestimate symptom burden in long-term head and neck cancer survivors.

## Introduction

The prevalence of long-term (5 to 10-year) survivors of oropharyngeal cancer has been increasing in recent years in the US [1]. Head and neck cancer, and its treatment, can lead to impairments in basic functions such as eating and speaking [2]. Symptoms may be present in long-term survivors because they developed around diagnosis or treatment and persisted or because they arose for the first time well after completion of treatment [3]. It is unclear how common symptoms are in long-term survivors [4]. Prior studies assessing symptom burden have generally included patients who were followed for at most 2–3 years after their cancer diagnosis [4]. A better understanding of the symptom burden faced by long-term survivors of head and neck cancer could improve patient care and help focus efforts on reducing toxicity from treatment.

A self-administered questionnaire is one potential source for assessing symptom burden in long-term head and neck cancer survivors [5,6,7,8,9]. Symptoms reported by head and neck cancer patients using the patient-reported outcome (PRO) version of the National Cancer Institute’s Common Terminology Criteria for Adverse Events (CTCAE) questionnaire were found to be more severe than those assessed by providers [10]. Yet, patient-reported symptoms may not be collected routinely in clinical settings with questionnaires. An alternative for research on long-term survivors may be diagnosis codes from health care encounters or provider notes from medical charts. However, the accuracy and completeness of these sources for assessing symptom burden is not clear. The purpose of the present study was twofold: 1) to describe the prevalence of symptoms in 5-year survivors of head and neck cancer using a self-administered questionnaire and, 2) to evaluate agreement between symptoms obtained via a patient self-administered questionnaire and two clinical data sources – provider notes from medical charts and diagnosis codes from health care encounters.

## Methods

### Setting and Sample Selection

All research activities were approved by the Kaiser Permanente Washington (KPWA) Institutional Review Board. This study was part of a broader study in which we identified all KPWA patients (n = 652) who had a diagnosis 5 years earlier (2011) of head and neck (the “index cancer”), lung or other cancers (e.g., thyroid, pituitary and skin of lips). Diagnoses were based on codes from the International Classification of Diseases for Oncology (ICD-O), 3rd Edition [11], and data were obtained from the linked KPWA and Surveillance, Epidemiology, and End Results program (SEER) Seattle-Puget Sound cancer registry [12]. We then excluded persons who were no longer enrolled in KPWA in 2016, were deceased, or were less than 18 years of age at diagnosis. Of the remaining 200 persons, we restricted the present analysis to those diagnosed with head and neck cancer. A total of 54 5-year survivors of head and neck cancer remained (see Appendix 1 for a list of ICD-O-3 codes).

### Self-Report Data Collection

All 5-year survivors (n = 54) were sent a letter that explained the purpose of the research and invited individuals to complete an online survey. By completing the survey, individuals provided consent to access their medical chart and claims information. To increase participation rates, each invitation included a $2 bill [13]. Invitees who had not completed the online survey after about 5 days were sent a reminder letter, a paper copy of the survey, and a postage-paid return envelope. If no response was received after an additional 5 days, a second paper survey was mailed. Persons who did not respond to the online or mailed surveys were contacted by phone to complete the survey. Everyone who completed the survey received $20 as a thank-you.

The survey included 78 questions about medical history, current health status, cancer symptom burden, lifestyle factors (e.g., smoking and use of alcohol), and demographic characteristics. For many questions, we used standardized items from published questionnaires [14,15,16]. Symptom burden within the past 24 hours was assessed using the MD Anderson Symptom Inventory head and neck cancer module (MDASI-HN) [17,18]. It includes 13 questions about symptoms related to cancer in general (“core symptoms”) and 9 questions about symptoms related to head and neck cancer specifically. Another six questions (“interference items”) address the degree to which a person’s overall symptom burden has interfered with aspects of their life (e.g., general activity, work, and mood). For each of the 28 questions, participants are asked to report the severity of the symptom (or interference) at its worst in the past 24 hours on a scale from 0 to 10 (“not present” to “as bad as you can imagine”).

### Clinical Data Collection

Data were also obtained on participants from two clinical sources: (1) provider notes in the electronic medical chart, and (2) administrative health plan data (i.e., diagnosis codes from health care encounters). Patient medical charts were reviewed during the 6-month period prior to completing the survey to assess the presence of a subset of symptoms from the MDASI-HN that consensus of the authors (which included clinicians who were informed by head and neck cancer patients) suggested were the most relevant to head and neck cancer patients: pain; mouth problems, such as dry mouth, mucus in the mouth and throat, altered or loss of taste, mouth or throat sores, and teeth or gum problems; difficulty with swallowing or chewing; aspiration of food or liquid; and difficulty with voice or speech. We chose a subset of the MDASI-HN symptom for medical record review because we wanted to focus study resources on the symptoms likely to be the most important to head and neck cancer patients. A trained medical chart abstractor assessed whether each symptom was present (yes/no) at any time during the 6-month period prior to the date the patient completed the self-report survey by reviewing the provider notes in the patient’s chart. ICD codes were not ascertained from the medical charts as this would have been redundant with the administrative data.

We also used administrative health plan data to assess symptom burden. We identified ICD-9/ICD-10 diagnosis codes that mapped to each symptom in the MDASI-HN (Appendix 2).

### Analyses

Descriptive statistics were used to characterize the study cohort. Sociodemographic information, cancer recurrence since 2011, current health status, and lifestyle factors were obtained from the patient survey. Index tumor information, first course of treatment, and additional demographic information (age at diagnosis, gender) were obtained from SEER registry.

We then computed the prevalence of each of the 22 symptoms from the MDASI-HN, along with their exact 95% binomial confidence intervals. Persons who rated the symptom as greater than 0 (no symptom) on a scale from 0–10 points were classified as having experienced the symptom. Persons who rated the symptom as 5 or greater were counted as having experienced the symptom with moderate or worse severity [19,20,21]. We used the same approach for the six MDASI-HN interference items.

For each symptom, we assessed the agreement of the patient’s survey response (the “gold standard”) with the (1) medical chart review and (2) administrative data. We computed the sensitivity (i.e., among patients who reported experiencing a given symptom in the survey, the percentage who also had evidence in the clinical source that they experienced that symptom), specificity (i.e., among patients who reported not experiencing a given symptom in the survey, the percentage who also lacked evidence in the clinical source that they experienced that symptom), positive predictive value (i.e., among patients with evidence in the clinical source that they experienced a given symptom, the percentage who also reported experiencing that symptom in the survey), and negative predictive value (i.e., among patients without evidence in the clinical source that they experienced a given symptom, the percentage who reported not experiencing that symptom in the survey, along with their exact 95 percent binomial confidence intervals, for each clinical data source. When the agreement estimate was 0 percent or 100 percent, a one-sided exact 97.5 percent binomial confidence interval was reported [22]. All analyses were conducted in Stata/MP 12.1.

## Results

### Study Population

Of the 54 5-year survivors of head and neck cancer eligible to participate, 43 (80 percent) completed the self-report survey: 12 online, 26 on paper, and 5 by phone. One person who reported in their survey to have never been diagnosed with head and neck cancer was excluded, which left 42 participants for analysis. The demographic characteristics of participants (n = 42) were similar to those eligible to participate (n = 54). Age at diagnosis 18–69 and ≥70 years was 74 percent and 26 percent, respectively, in both groups. Participants were slightly more likely than those eligible to be male (74 percent versus 68 percent), and a race other than white (14 percent versus 7 percent).

Characteristics of the participants are described in Table 1. Stage at diagnosis was I or II for 58 percent of participants and III or IV for 42 percent. Mean age at diagnosis was 62 years. Participants tended to be male (74 percent) and report white race (86 percent). It was uncommon for participants to report being a current smoker (5 percent); however, most were former smokers (59 percent). A total of 62 percent of participants reported current alcohol use.

**Table 1:** Characteristics of 5-year survivors of head and neck cancer diagnosed in 2011 at Kaiser Permanente Washington (n = 42).

	n	%

Cancer site		
Aerodigestive	38	90.4
Tongue	13	30.9
Gum, floor of mouth, and other mouth	9	21.4
Tonsil	6	14.3
Larynx	6	14.3
Lip, nasopharynx, and salivary gland	4	9.5
Non-aerodigestive^a^	4	9.5
AJCC stage^b^		
I	16	42.1
II	6	15.8
III	6	15.8
IV	10	26.3
Unknown	4	
Treatment		
Surgery only^c^	15	35.7
Surgery and radiation only	8	19.0
Surgery, radiation and chemotherapy	5	11.9
Radiation only	3	7.1
Radiation and chemotherapy only	11	26.2
Self-reported cancer recurrence		
Yes	11	26.8
No	30	73.2
Missing	1	
Self-reported time since last cancer treatment		
<3 years ago	11	27.5
≥3 years ago	29	72.5
Missing	2	
At least one in-person visit with a provider in the prior 6 months		
Yes	35	87.5
No	5	12.5
Missing	2	
Age at index cancer diagnosis (years)		
18-49	5	11.9
50-69	26	61.9
≥70	11	26.2
Gender		
Female	11	26.2
Male	31	73.8
Race^d^		
White	36	85.7
Other	6	14.3
Current marital status^e^		
Married or living with a partner	35	85.4
Other	6	14.6
Missing	1	
Current highest level of education^e^		
High school or less	11	26.2
Technical school or some college	13	31.0
4-year college or postgraduate degree	18	42.9
Current occupational status^e^		
Employed or homemaker	17	40.5
Unemployed, retired or disabled	25	59.5
Current annual household income^e^		
<$50,000	12	30.8
$50,000–<$100,000	13	33.3
≥$100,000	14	35.9
Missing	3	
Current self-reported general health status^e^		
Excellent	6	14.6
Very good	14	34.1
Good	14	34.1
Fair or poor	7	17.1
Missing	1	
Current self-reported smoking status^e^		
Never	15	36.6
Former	24	58.5
Current	2	4.9
Missing	1	
Current self-reported alcohol use^e^		
None	16	38.1
<2–3 times per week	11	26.2
≥2–3 times per week	15	35.7

^a^ Lymph nodes of head, face, and neck; thymus; and peripheral nerves and autonomic nervous system of head, face and neck. ^b^ American Joint Committee on Cancer. ^c^ Includes one patient with missing information on receipt of radiation therapy. ^d^ Hispanic ethnicity was not present (n = 40) or missing (n = 2). ^e^ Current refers to the time the patient completed the survey in 2016.

### Symptom prevalence in 5-year survivors of head and neck cancer

Nearly all participants (95 percent) reported experiencing at least one symptom from the MDASI-HN, and 93 percent reported two or more symptoms. The majority of participants had dry mouth, fatigue, drowsiness, problems with tasting food, and difficulty remembering (range 58 percent–69 percent, Table 2). About half of participants (46 percent–54 percent) had distress, sadness, numbness/tingling, difficulty with swallowing/chewing, problems with teeth or gums, problems with mucus in the mouth/throat, disturbed sleep, coughing/choking, and difficulty with voice/speech. A quarter to less than half of participants (24 percent–39 percent) had constipation, pain, lack of appetite, shortness of breath, skin pain/burning/rash, and mouth/throat sores. Nausea was reported by 20 percent of participants and vomiting by 10 percent.

**Table 2:** Distribution of MDASI-HN symptom and interference scores (possible range: 0–10) among 5-year survivors of head and neck cancer diagnosed in 2011 at Kaiser Permanente Washington (n = 42).

MDASI-HN item	Mean score	95% CI	Median (IQR)	Range	% any severity (score ≥ 1)		% moderate or greater severity (score ≥ 5)	

					n	% (95% CI)	n	% (95% CI)

**Core symptom**								

Dry mouth	4.1	3.0–5.3	4 (0–8)	0–10	29	69 (53–82)	21	50 (34–66)
Fatigue (tiredness)	2.9	2.1–3.8	2 (0–5)	0–10	27	64 (48–78)	13	31 (18–47)
Drowsy (sleepy)^a^	2.1	1.3–2.8	1 (0–3)	0–8	25	61 (45–76)	7	17 (7–32)
Difficulty remembering	2.0	1.2–2.7	1 (0–3)	0–8	24	57 (41–73)	7	17 (7–31)
Being distressed (upset)^a^	1.9	1.1–2.8	1 (0–3)	0–10	22	54 (37–69)	7	17 (7–32)
Sadness^a^	2.1	1.3–3.0	1 (0–4)	0–9	22	54 (37–69)	8	20 (9–35)
Numbness/tingling^a^	2.0	1.2–2.8	1 (0–4)	0–8	22	54 (37–69)	8	20 (9–35)
Disturbed sleep^a^	2.1	1.2–2.9	0 (0–3)	0–9	19	46 (31–63)	7	17 (7–32)
Lack of appetite^a^	1.7	0.8–2.5	0 (0–3)	0–10	16	39 (24–55)	7	17 (7–32)
Pain	1.9	1.0–2.9	0 (0–3)	0–10	16	38 (24–54)	9	21 (10–37)
Shortness of breath	1.4	0.6–2.2	0 (0–2)	0–10	15	36 (22–52)	5	12 (4–26)
Nausea^a^	0.7	0.1–1.3	0 (0–0)	0–8	8	20 (9–35)	3	7 (2–20)
Vomiting^a^	0.1	0.0–0.2	0 (0–0)	0–1	4	10 (3–23)	0	0 (0–9)^c^
**Head and neck symptom**								

Problem with tasting food^b^	2.8	1.8–3.8	2 (0–5)	0–10	23	58 (41–73)	12	30 (17–47)
Difficulty with swallowing/chewing^a^	2.8	1.7–3.9	1 (0–5)	0–10	22	54 (37–69)	6	15 (6–29)
Problem with teeth or gums^a^	2.4	1.4–3.4	1 (0–3)	0–10	22	54 (37–69)	10	24 (12–40)
Problem with mucus in mouth/throat^a^	2.3	1.4–3.3	1 (0–4)	0–10	21	51 (35–67)	9	22 (11–38)
Coughing/choking	2.1	1.1–3.0	0 (0–3)	0–10	19	46 (31–63)	8	20 (9–35)
Difficulty with voice/speech^a^	2.0	1.0–2.9	0 (0–4)	0–10	19	46 (31–63)	6	15 (6–29)
Constipation^a^	1.7	0.9–2.6	0 (0–3)	0–9	18	44 (28–60)	6	15 (6–29)
Skin pain/burning/rash^b^	1.2	0.4–2.0	0 (0–1)	0–9	10	25 (13–41)	6	15 6–29)
Mouth/throat sores^a^	1.4	0.5–2.3	0 (0–0)	0–10	10	24 (12–40)	8	20 (9–35)
**Interference**								

Enjoyment of life^a^	2.5	1.5–3.6	1 (0–5)	0–10	23	56 (26–57)	11	27 (14–43)
Activity^b^	2.2	1.3–3.0	2 (0–4)	0–10	21	53 (36–69)	6	18 (7–33)
Work^a^	2.3	1.4–3.2	1 (0–5)	0–10	21	51 (35–67)	11	27 (14–43)
Mood^b^	1.8	1.0–2.6	0 (0–3)	0–10	19	48 (31–64)	6	15 (6–30)
Relations with others^a^	1.7	0.9–2.4	0 (0–2)	0–9	19	46 (30–62)	6	15 (6–29)
Walking^a^	2.1	1.1–3.1	0 (0–3)	0–10	17	42 (26–57)	9	22 (11–38)

CI = confidence interval; MDASI-HN = MD Anderson Symptom Inventory head and neck cancer module. ^a^ Based on the 41 patients who responded to this item. ^b^ Based on the 40 patients who responded to this item. ^c^ One-sided 97.5% confidence interval.

Overall, 71 percent of participants reported experiencing at least one symptom from the MDASI-HN that was of moderate or worse severity, and 58 percent reported two or more symptoms. The most common symptom of moderate or worse severity was dry mouth (50 percent) (Table 2).

Approximately half of participants reported that their symptoms interfered to some degree with the domains of life examined in the survey (range 42 percent–56 percent, Table 2), and 15 percent–27 percent of participants reported that their symptoms interfered moderately or worse with at least one of the domains. Enjoyment of life, work, and activity were the most common domains to be affected.

### Agreement between self-report and clinical data sources

#### Medical Charts

For the nine MDASI-HN symptoms (of a total of 22) selected because of their likely importance to head and neck cancer patients, we assessed the agreement of symptom ascertainment via medical chart review, with the participant’s survey response serving as the gold standard (Table 3). In general, the medical chart was specific (i.e., a relatively high percentage of patients who reported not experiencing a given symptom in the survey also lacked evidence of that symptom in the medical chart) but not sensitive (i.e., a relatively low percentage of patients who reported experiencing a given symptom in the survey also had evidence of that symptom in the medical chart); pain was the only symptom for which sensitivity was higher than specificity. For example, among patients who reported having a symptom, the presence of the symptom was also recorded in the medical chart (sensitivity) in 4 percent–36 percent of those patients, depending on the symptom, except for pain, which was recorded in the chart in 75 percent of them. And among patients who reported they did not have a symptom, the symptom was also absent in the chart (specificity) in 90 percent–100 percent of those patients, depending on the symptom, except for pain, which was also absent in the chart in only 46 percent of them.

**Table 3:** Accuracy of ascertainment of head and neck cancer symptoms from medical chart review and administrative data as compared to a patient survey (“gold standard”) among 5-year survivors of head and neck cancer diagnosed in 2011 at Kaiser Permanente Washington (n = 42).

Symptom	Prevalence (Survey)^f^		Medical Chart^g^ vs. Survey				Administrative Data^h^ vs. Survey			

	n	% (95% CI)	Sensitivity (95% CI)	Specificity (95% CI)	PPV (95% CI)	NPV (95% CI)	Sensitivity (95% CI)	Specificity (95% CI)	PPV (95% CI)	NPV (95% CI)

**MDASI-HN item^d^**										

Dry mouth	29	69 (53–82)	21 (8–40)	100 (75–100)	100 (54–100)	36 (54–100)	7 (1–23)	100 (75–100)	100 (16–100)	33 (19–49)
Problem with tasting food^b^	23	58 (41–73)	4 (0–22)	100 (81–100)	100 (3–100)	44 (28–60)	0 (0–15)^c^	100 (80–100)^c^	undefined^e^	41 (26–58)
Difficulty with swallowing/chewing^a^	22	54 (37–69)	36 (17–59)	95 (74–100)	89 (52–100)	56 (38–74)	23 (8–45)	90 (67–99)	71 (29–96)	50 (32–68)
Problem with teeth or gums^a^	22	54 (37–69)	23 (8–45)	100 (82–100)	100 (48–100)	53 (36–70)	5 (37–69)	95 (74–100)	50 (1–99)	46 (30–63)
Problem with mucus in mouth and throat^a,i^	21	51 (35–67)	19 (5–42)	95 (75–100)	80 (28–100)	53 (36–70)	N/A	N/A	N/A	N/A
Coughing/choking	19	46 (31–63)	11 (1–3)	100 (85–100)	100 (16–100)	56 (40–72)	16 (3–40)	86 (65–97)	50 (12–88)	54 (37–71)
Difficulty with voice/speech^a^	19	46 (31–63)	26 (9–51)	96 (77–100)	83 (36–100)	60 (42–76)	0 (0–18)^c^	100 (85–100)^c^	undefined^e^	54 (37–69)
Pain	16	38 (24–54)	75 (48–93)	46 (27–67)	46 (27–67)	75 (48–93)	38 (15–65)	85 (65–96)	60 (26–88)	69 (50–84)
Mouth/throat sores^a^	10	24 (12–40)	20 (3–56)	90 (74–98)	40 (5–85)	78 (61–90)	40 (12–74)	94 (79–99)	67 (22–96)	83 (66–93)

^a^ Based on the 41 patients who responded to this item. ^b^ Based on the 40 patients who responded to this item. ^c^ One-sided 97.5% confidence interval. ^d^ For the MDASI-HN survey items, the symptom was considered absent if the patient’s score was 0 and present if it was ≥1. ^e^ Denominator was equal to 0. ^f^ Patients were asked about their symptoms at their worst in the past 24 hours. ^g^ Patient information was ascertained from the medical chart for the 6 month period prior to completion of the survey. ^h^ Patient information was ascertained from administrative data for the 6 month period prior to completion of the survey. ^i^ Agreement with administrative data could not be determined because we did not identify ICD-9 and ICD-10 codes that mapped to this symptom.

Among patients with a record of the symptom in the medical chart, the symptom was also reported in the survey (positive predictive value [PPV]) in 83 percent–100 percent of those patients, depending on the symptom, except for pain and mouth/throat sores, which had lower PPVs. Among patients without a record of the symptom in the medical chart, the symptom was also reported to not be present in the survey (negative predictive value [NPV]) in 36 percent–78 percent of those patients, depending on the symptom.

#### Administrative Data

For eight of the nine selected MDASI-HN symptoms assessed by medical chart review, we also assessed the agreement of symptom ascertainment via administrative data, with the participant’s survey response serving as the gold standard (Table 3). The sensitivity of administrative data for symptom ascertainment was generally lower than medical chart review, but the range was similar (0 percent–40 percent). The specificity of the administrative data was similar to medical chart review; it ranged from 85 percent–100 percent, depending on the symptom. The PPV of the administrative data could not be computed for two of the eight symptoms (problems with tasting food and difficulty with voice/speech) because we did not identify any participants with these symptoms through the use of diagnosis codes, even though the prevalence of these symptoms (based on survey responses) was appreciable (58 percent and 46 percent, respectively). For the remaining six of the eight symptoms, the PPV of the administrative data ranged from 50 percent–100 percent. The NPV of the administrative data was similar to medical chart review; it ranged from 33 percent–83 percent, depending on the symptom.

For the remaining thirteen MDASI-HN symptoms (not assessed by medical chart review), we assessed the agreement of symptom ascertainment with administrative data only (Table 4). The sensitivity was generally low, ranging from 0 percent–32 percent. Specificity ranged from 91 percent–100 percent. The PPV could not be computed for two symptoms—drowsiness (prevalence from survey = 61 percent) and lack of appetite (prevalence from survey = 39 percent)—as we did not identify any participants with these symptoms using diagnosis codes. For the remaining eleven of the thirteen symptoms, the PPV was 71 percent–100 percent, depending on the symptom, except for skin pain/burning/rash, constipation, vomiting, nausea and shortness of breath, which each had a PPV less than 50 percent. The NPV ranged from 38 percent–74 percent depending on the symptom.

**Table 4:** Accuracy of ascertainment of head and neck cancer symptoms from administrative data as compared to a patient survey (“gold standard”) among 5-year survivors of head and neck cancer diagnosed in 2011 at Kaiser Permanente Washington (n = 42).

Symptom	Survey Prevalence^d^		Administrative Data^e^ vs Survey			

	n	%(95% CI)	Sensitivity (95% CI)	Specificity (95% CI)	PPV (95% CI)	NPV (95% CI)

**MDASI-HN item^c^**						

Fatigue (tiredness)	27	64 (48–78)	7 (1–24)	100 (78–100)	100 (16–100)	38 (23–54)
Drowsy (sleepy)^a^	25	61 (45–76)	0 (0–14)^e^	100 (79–100)^e^	undefined	38 (24–54)
Remembering things	24	57 (41–73)	4 (0–21)	100 (82–100)	100 (3–100)	44 (29–60)
Being distressed (upset)^a^	22	54 (37–69)	32 (14–55)	95 (74–100)	88 (47–100)	55 (36–72)
Sadness^a^	22	54 (37–69)	32 (14–55)	100 (82–100)	100 (59–100)	56 (38–73)
Numbness/tingling^a^	22	54 (37–69)	5 (0–23)	100 (82–100)	100 (3–100)	48 (32–64)
Disturbed sleep^a^	19	46 (31–63)	26 (9–51)	91 (71–99)	71 (29–96)	59 (41–75)
Constipation^a^	18	44 (28–60)	6 (0–27)	91 (72–99)	33 (1–91)	55 (38–71)
Lack of appetite^a^	16	39 (24–55)	0 (0–21)^f^	100 (86–100)^f^	undefined^g^	60 (43–74)
Shortness of breath	15	36 (22–52)	13 (2–41)	93 (76–99)	50 (7–93)	66 (49–80)
Skin pain/burning/rash^b^	10	25 (13–41)	0 (0–31)	98 (83–100)	0 (0–98)	74 (58–87)
Nausea^a^	8	20 (9–35)	13 (0–53)	94 (80–99)	33 (1–91)	82 (66–92)
Vomiting^a^	4	10 (3–23)	0 (0–60)	97 (86–100)	0 (0–97.5)	90 (76–97)

^a^ Based on the 41 patients who responded to this item. ^b^ Based on the 40 patients who responded to this item. ^c^ For the MDASI-HN survey items, the symptom was considered absent if the patient’s score was 0 and present if it was ≥1. ^d^ Patients were asked about their symptoms at their worst in the past 24 hours. ^e^ Patient information was ascertained from administrative data for the 6 month period prior to completion of the survey. ^f^ One-sided 97.5% confidence interval. ^g^ Denominator was equal to 0.

We additionally assessed the agreement of both data sources when the moderate or worse cut-point was used to classify the patient as having the symptom; a symptom was considered absent if the MDASI-HN score was <5 and present if it was ≥5. The use of this different cut-point did not appreciably alter our findings (data not shown). We also compared agreement when information from both clinical sources was combined. We found no appreciable differences in our results; there were only slight increases in sensitivity and slight decreases in specificity for some symptoms (data not shown).

## Discussion

Among long-term survivors of head and neck cancer who were included in this study, we found that nearly all had one or more of the 22 MDASI-HN symptoms five years after their diagnosis. A total of 93 percent had two or more symptoms, and 71 percent had one or more symptoms which they rated as being more than mild in severity (i.e., moderate or severe). For approximately one in four of the 5-year survivors, their symptom(s) interfered moderately or severely with their enjoyment of life and, for a similar proportion, interfered moderately or severely with their work.

We also found poor agreement between symptoms ascertained from the clinical data sources (i.e., diagnosis codes from health care encounters and provider notes from medical charts) and patient self-report. Overall, when compared to patient self-report, we found that the clinical sources had low sensitivity and moderate to high specificity. PPV tended to be high and NPV was low to moderate, depending on the symptom. In general, the sensitivity of the medical chart was better than the administrative data, but our results indicate that relying on either clinical source, alone or in combination, would lead to appreciable underestimation of symptom prevalence.

The one notable discrepancy to this observation was pain. For pain, sensitivity of the medical chart was relatively high (75 percent) and specificity was relatively low (46 percent). Possible reasons for the relatively high sensitivity are that providers may have been more likely to ask patients about pain compared to other symptoms, and patients may have been more likely to report pain compared to other symptoms they were experiencing.

Several study limitations are worth noting. First, our sample of 5-year head and neck cancer survivors was small (n = 42), which contributed to somewhat imprecise estimates of symptom prevalence and agreement measures. Symptom prevalence may vary according to stage at diagnosis and treatment received but we did not have sufficient sample size to meaningfully evaluate differences. Additionally, the time frame for symptom ascertainment did not completely align between the patient self-report (previous 24 hours) and the clinical data sources (previous 6 months). Although symptom severity assessed using the MDASI-HN has been found to be similar when a 7-day recall period was used compared to a 24-hour period [23], patients’ symptoms during the 6 months before the survey may have resolved by the time the survey was completed.

As mentioned above, we observed low sensitivity of clinical data sources (both the medical chart and administrative data) for symptom ascertainment. The lack of symptom information in the clinical sources may have been due to the patient not seeking care, the patient not mentioning the symptom to the provider, the provider not asking the patient about a symptom, and/or the provider not documenting the symptom in the clinical record. It is also possible that we missed some relevant diagnosis codes that were used by providers to record symptoms, or that relevant diagnosis codes did not exist. Indeed, we observed that for four relatively common symptoms—problems tasting food, difficulty with voice/speech, drowsiness, and lack of appetite (range in prevalence was 39 percent–61 percent based on self-report)—there was no patient who had evidence of any of these symptoms in the administrative data. And for one symptom, problems with mucus in the mouth or throat, no relevant diagnosis code was identified.

An important strength of our study was the high proportion of patients who responded to the survey (80 percent). As such, it is unlikely that selection bias meaningfully influenced our conclusions.

In conclusion, we observed an appreciable symptom burden in long-term head and neck cancer survivors. Further, relying on clinical data alone substantially underestimates patients’ symptom burden compared to self-report. These findings suggest that clinical sources (i.e., diagnosis codes from health care encounters and medical chart review) should not be used to estimate symptom prevalence in long-term head and neck cancer survivors.

## Additional Files

The additional files for this article can be found as follows:

10.5334/egems.271.s1 Appendix 1. Head and neck cancer sites used to identify 5-year survivors of head and neck cancer diagnosed at Kaiser Permanente Washington in 2011.

10.5334/egems.271.s2 Appendix 2. Diagnosis codes (ICD-9 and ICD-10) used to identify symptoms from the MDASI-HN questionnaire.
