# Supplementary material for: Symptom Burden in Long-Term Survivors of Head and Neck Cancer: Patient-Reported Versus Clinical Data
Source: EGEMS (Wash DC). 2019 Jul 10;7(1):25. doi: 10.5334/egems.271 (PMC6625536; doi:10.5334/egems.271)
Supplement: Appendix 1. — Head and neck cancer sites used to identify 5-year survivors of head and neck cancer diagnosed at Kaiser Permanente Washington in 2011. [file egems-7-1-271-s1.pdf]

Appendix 1. Head and neck cancer sites used to identify 5-year survivors of head and neck cancer diagnosed at Kaiser Permanente Washington in 2011.

Site	ICD-O-3 site code[a]
**Salivary gland**	C079-C089
**Anterior skull base**	
Nose, nasal cavity, and paranasal sinuses	C300, C310-C319
**Lateral skull base**	
Cranial nerve	C725
**Aerodigestive**	
Lip	C000-C009
Tongue	C019-C029
Floor of Mouth	C040-C049
Gum and Other Mouth	C030-C039, C050-C059, C060-C069
Nasopharynx	C110-C119
Tonsil	C090-C099
Oropharynx	C100-C109
Hypopharynx	C129, C130-C139
Other Oral Cavity and Pharynx	C140, C142, C148
Larynx	320-C329
Cervical esophagus	C150
Trachea	C339
**Bone and soft tissue**	
Soft tissues of head face and neck	C490
Bones of skull and face	C410
Mandible	C411
**Other**	
Lymph nodes of head, face and neck	C770
Thymus	C379
Carotid body	C754
Peripheral nerves and autonomic nervous system of head face neck	C470

[a]All histologies except 9050-9055, 9140, and 9590-9992 were included for all sites, with the exception of lymph nodes of head, face and neck, wherein histologies 9650-9667, 9590-9597, 9670-9671, 9673, 9675, 9678-9680, 9684, 9687-9691, 9695, 9698-9702, 9705, 9708-9709, 9712, 9714-9719, 9724-9729, 9735, 9737-9738, 9811-9818, 9823, 9827, 9837 were not included.
