# Supplementary material for: Symptom Burden in Long-Term Survivors of Head and Neck Cancer: Patient-Reported Versus Clinical Data
Source: EGEMS (Wash DC). 2019 Jul 10;7(1):25. doi: 10.5334/egems.271 (PMC6625536; doi:10.5334/egems.271)
Supplement: Appendix 2. — Diagnosis codes (ICD-9 and ICD-10) used to identify symptoms from the MDASI-HN questionnaire. [file egems-7-1-271-s2.pdf]

Appendix 2. Diagnosis codes (ICD-9 and ICD-10) used to identify symptoms from the MDASI-HN questionnaire.

| Symptoms | ICD-9 code | ICD-10 code |
|---|---|---|
| **Included in Table 3** | | |
| Dry mouth | 527.7 | K11.7, R68.2 |
| Problem with tasting food | 781.1, V41.5 | R43.x |
| Difficulty with swallowing/ chewing | 787.20, 787.21, 787.22, 787.23, 787.24, 787.29 | R13.0, R13.10, R13.11, R13.12, R13.13, R13.14, R13.19, V41.6 |
| Problem with teeth or gums | 521.0x, 522.x, 523.xx | K02.x, K04.x, K05.x, K06.x |
| Problem with mucus in mouth and throat | No relevant code identified | No relevant code identified |
| Coughing/choking | 786.2, 786.93, 784.99, 933.0, 933.1, 934.0, 934.1, 934.8, 934.9, E911 | R05, R04.2, T17.200x, T17.210x, T17.220x, T17.300x, T17.310x, T17.320x, T17.400x, T17.410x, T17.420x, T17.500x, T17.510x, T17.520x, T17.800x, T17.810x, T17.820x, T17.900x, T17.910x, T17.920x |
| Difficulty with voice/speech | 784.41, 784.51, 784.59, 478.30, 478.31, 478.32, 478.33, 478.34, 478.79, V41.4 | R47.1, R47.81, R47.89, R47.9, R49.1, R49.8, R49.9, J38.00, J38.01, J38.02, J38.7 |
| Pain | Site not specified<br>307.80, 307.89, 338.0, 338.11, 338.12, 338.18, 338.19, 338.21, 338.22, 338.28, 338.29, 338.3, 338.4, 780.96, 729.1, 729.2<br>Ear<br>388.70, 388.71, 388.72<br>Eye<br>379.91<br>Face<br>784.0<br>Throat<br>784.1<br>Jaw<br>784.92<br>Chest<br>786.50, 786.51, 786.52, 786.59<br>Neck<br>723.1, 723.3, 723.4, 723.9<br>Shoulder<br>719.41, 719.61, 719.91 | Site not specified<br>F45.41, F45.42, G89.0, G89.11, G89.12, G89.18, G89.21, G89.22, G89.28, G89.29, G89.3, G89.4, R52, M60.80, M60.88, M60.89, M60.9, M79.1, M79.2, M79.7, R54.10<br>Ear<br>H92.0x<br>Eye<br>H57.1x<br>Face<br>R51<br>Throat<br>R07.0<br>Jaw<br>R68.84<br>Chest<br>R07.1, R07.2, R07.81, R07.82, R07.89, R07.9, M54.14, M54.15, M54.16, M54.17<br>Neck<br>M54.2, M53.0, M53.1, M54.11, M54.12, M54.13, M53.82 |

| | | |
|---|---|---|
| | | Shoulder<br>M25.51x, R29.898, M25.9 |
| Mouth/throat sores[a] | 528.9, 528.00, 528.01, 528.02, 528.09, 528.6, 528.79, 702.8 528.2, 701.5, 054.73 | K12.0, K12.1, K12.2, K12.3x, K13.2x, K13.3, K13.4, K13.6, K13.7x, B00.1 |
| **Included in Table 4** | | |
| Fatigue (tiredness) | 780.79, 300.5 | R53.0, R53.1, R53.8x, F48.8 |
| Drowsy (sleepy) | 780.09, 307.43, 307.44 | R40.0, F51.11, F51.19 |
| Remembering things | 780.93, 331.83 | R41.2, R41.3, G31.84, R41.81 |
| Being distressed (upset) | 300.00, 300.01, 300.02, 300.09, 293.84, 309.0, 309.24, 291.89 | F41.x, F06.4, F43.x, F10.159, F10.180, F10.280, F10.980, F12.180, F12.280, F12.980, F13.180, F13.280, F13.980, F14.180, F14.280, F14.980, F16.180, F16.280, F16.980, F18.180, F18.280, F18.980, F19.180, F19.280, F19.980 |
| Sadness | 311, 296.20, 296.21, 296.22, 296.23, 296.24, 296.25, 296.30, 296.31, 296.32, 296.33, 296.34, 296.35, 296.82, 298.0, 300.4, 301.12, 309.0, 309.1, 309.28, 293.83 | F06.31, F06.32, F32.0, F32.1, F32.2, F32.3, F32.4, F32.8, F32.9, F33.0, F33.1, F33.2, F33.3, F33.40, F33.41, F33.8, F33.9, F34.1, F43.21, F43.23 |
| Numbness/tingling | 357.3, 356.4, 356.8, 356.9, 355.9 | G63, G60.3, G60.9, G60.9, G58.9, R20.0, R20.2 |
| Disturbed sleep | 780.50, 780.51, 780.52, 780.53, 780.57, 780.58, 307.40, 307.41, 307.42, 307.49, 327.00, 327.01, 327.02, 327.09, 327.20, 327.21, 327.23, 327.24, 327.26, 327.27, 327.29, 333.94, 291.82, 292.85 | G47.6x, G47.8, G47.9, G47.30, G47.31, G47.33, G47.34, G47.36, G47.37, G47.39, G47.00, GT47.01, G47.09, F51.8, F51.9, F51.01, F51.02, F51.03, F51.04, F51.05, F51.09, G25.81, F10.182, F10.282, F10.982, F11.182, F11.282, F11.982, F13.182, F13.282, F13.982, F14.182, F14.282, F14.982, F15.182, F15.282, F15.982, F19.182, F19.282, F19.982 |
| Constipation | 564.00 | K59.0x |
| Lack of appetite | 783.0, 307.50, 307.59 | R63.0, F50.8, F50.9 |
| Shortness of breath | 786.00, 786.02, 786.05, 786.09 | R06.00, R06.01, R06.02, R06.09 |
| Skin pain/burning/rash | 782.0, 782.1 | R20.1, R20.3, R20.8, R20.9, R21 |
| Nausea | 787.01, 787.02 | R11.0, R11.2 |
| Vomiting | 787.03, 787.04, 578.0, 307.54, 564.3 | R11.1x, G43.Ax, K92.0, F50.8, K91.0 |
